# 

**Published:** 1919-02-08

**Authors:** 


### THE COLLEGE OF NURSING (Limited by Guarantee)

### THE YORKSHIRE CENTRE.

(Hon. Sec., Miss M. V. LINDALL, Hospital for Women and Children, Leeds.)

THE next meeting will be held at the Leeds General Infirmary, Clinical Theatre, on Thursday, February 13, at 6 P.M., when Captain M. J. Stewart, M.B., M.R.C.P., will lecture on "The Defences of the Body Against Infection." All nurses are cordially invited. Fee, 1s. each lecture to non-members.

### MANCHESTER CENTRE.

(Hon. Sec., Miss MAUD EARL, Ancoats Hospital, Manchester.)

A GENERAL meeting will be held on Friday, February 21, at the Albert Hall, Peter Street, commencing at 7 P.M. All members are cordially invited.

A whist drive was held on January 29 for members of the local Centre, and in spite of bad weather and the fact that many of the members live some way out of Manchester, there was a large number present, and a very enjoyable evening was spent.

### Forthcoming Events.

Medical Society of London.—The following is the programme for the second half of the present season: February 10, paper on "Intrinsic Cancer of the Larynx: Operation by Larynx-Fissure and its results," Sir St. Clair Thomson, M.D. February 24, paper on the "Treatment of Congenital Hypertrophy of the Pylorus," Mr. Robert Anstruther Ramsay, M.C. March 10, 17, and 24, the Lettsomian Lectures on "Jaundice" will be delivered by Col. W. H. Willcox, C.B., C.M.G., A.M.S., M.D. April 14, Clinical Evening. May 12, General Meeting, followed at 9 p.m. by The Annual Oration to be delivered by Sir John Tweedy, LL.D., F.R.C.S.

